# Modulating superabsorbent polymer properties by adjusting the amphiphilicity

**DOI:** 10.3389/fchem.2022.1009616

**Published:** 2022-09-13

**Authors:** Craig W. Stocker, Maoqi Lin, Vanessa N. L. Wong, Antonio F. Patti, Gil Garnier

**Affiliations:** ^1^ Department of Chemical and Biological Engineering, Bioresource Processing Research Institute of Australia (BioPRIA), Monash University, Clayton, VIC, Australia; ^2^ School of Earth, Atmosphere and Environment, Monash University, Clayton, VIC, Australia; ^3^ School of Chemistry, Monash University, Clayton, VIC, Australia

**Keywords:** crosslinking, superabsorbent, amphiphilic, swelling, hornification, cellulose

## Abstract

The role of amphiphilicity in polysaccharide-based superabsorbent polymers is paramount in determining material properties. While the performance of freeze-dried polymers is improved by maximizing hydrophilicity, this may not be the case for evaporative-dried polymers. In this study, four diglycidyl ether crosslinkers, with varying chain lengths and amphiphilicities, were used to synthesize a series of evaporative-dried carboxymethyl cellulose-based superabsorbent films. Through structural and physiochemical characterization, the effect of amphiphilicity on swelling and mechanical properties was established. Contrary to freeze-dried polymers, it was found that the addition of hydrophobic moieties by crosslinking with novel poly(propylene glycol) diglycidyl ether crosslinkers increased the swelling performance of evaporative-dried polymers. By adding hydrophobic functional groups, a reduction in inter-chain hydrogen bonding occurs during evaporative-drying, reducing the degree of hornification and decreasing the entropy requirement for water uptake. By optimizing the amphiphilic ratio, a poly(propylene glycol)-carboxymethyl cellulose polymer achieved a swelling capacity of 182 g/g which is competitive with freeze-dried cellulose-based hydrogels. The mechanical properties of these films improved with the addition of the crosslinkers, with glycerol-carboxymethyl cellulose polymers achieving a tensile strength of 39 MPa and a Young’s Modulus of 4.0 GPa, indicating their potential application as low-cost, swellable films.

## 1 Introduction

Superabsorbent polymers are materials that can absorb hundreds of times their own weight in water while remaining stable (Mendoza et al., 2019a). As the world progresses towards a renewable future, cellulose is becoming increasingly popular as a platform molecule for superabsorbent materials due to its low-cost, biocompatibility, and ability to modify for desired applications (Alam et al., 2019; Mendoza et al., 2019a; Barajas-Ledesma et al., 2020; Zainal et al., 2021). In our previous work we have investigated the synthesis and properties of TEMPO-oxidized cellulose-based hydrogels for agricultural and biomedical applications (Mendoza et al., 2019b; Curvello et al., 2019; Barajas-Ledesma et al., 2020; Hossain et al., 2021; Barajas-Ledesma et al., 2022).

Typically, cellulose is chemically modified (Saito et al., 2007; Mendoza et al., 2018; Mendoza et al., 2019a; Filipova et al., 2020) and often crosslinked (Cuadro et al., 2015; Capanema et al., 2018; Alam et al., 2019) to improve mechanical properties and create a stable porous structure. However, a challenge with using cellulose and other polysaccharides for the synthesis of superabsorbent materials is the reduced performance after evaporative drying. This is caused by the irreversible agglomeration of adjacent polysaccharide chains through the formation of interchain hydrogen bonds, also known as hornification for cellulose-based materials. This causes the material to shrink and deform, reducing mechanical strength (Butchosa and Zhou, 2014; Salmén and Stevanic, 2018; Islam et al., 2019) and collapsing the pore structure, thereby inhibiting swelling properties (Barajas-Ledesma et al., 2020). Many studies have used freeze-drying as an alternative method to preserve the structure of polysaccharide-based polymers while drying; however, this method is expensive and not scalable (Kono, 2014; Mendoza et al., 2019a).

Other methods investigated to counter this phenomenon include chemical modification by carboxylation to reduce hornification by electrostatic stabilization (Barajas-Ledesma et al., 2020), and the addition of plasticizers such as polyethylene glycol (PEG) to inhibit the formation of interchain hydrogen bonds (Johns et al., 2021; Ucpinar Durmaz and Aytac, 2021).

**FIGURE 1:**
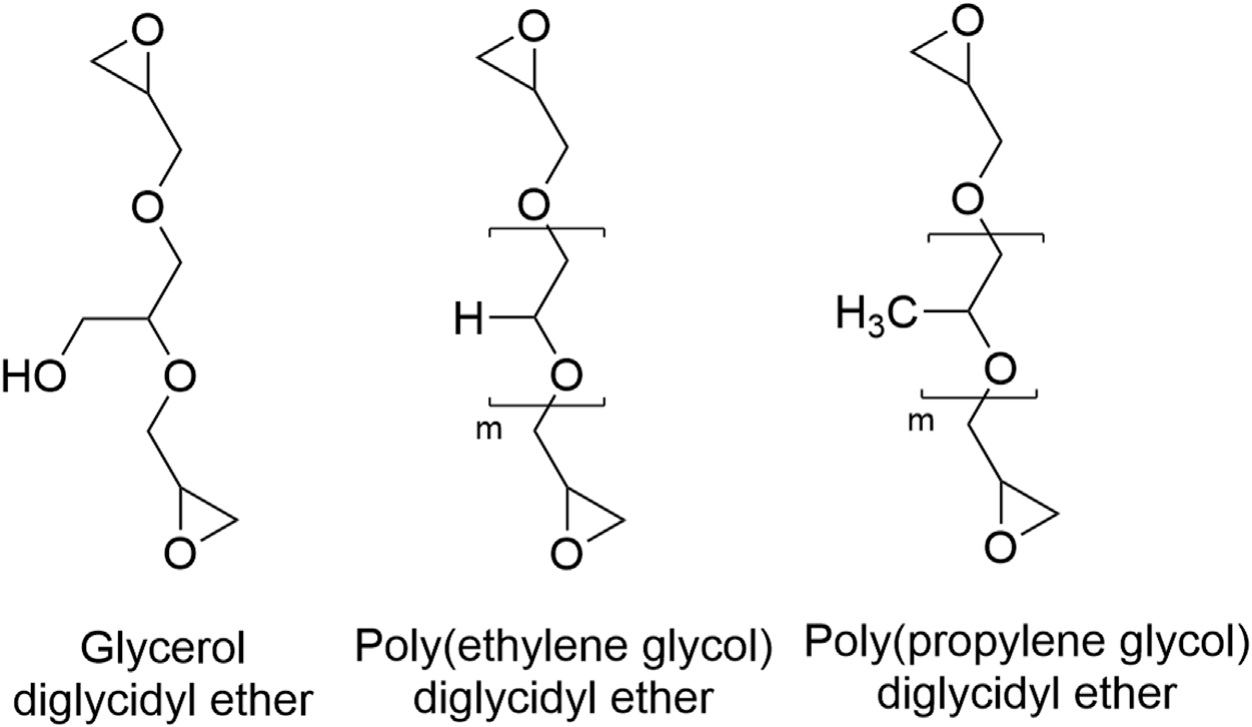
Chemical structure of the three diglycidyl ethers investigated as crosslinkers.

Of interest, however, is the effect of crosslinker chain length and amphiphilicity through the application of various diglycidyl ethers (DEs) (Figure 1) as dual-purpose crosslinkers and plasticizing agents for the optimization of evaporative-dried cellulose-based superabsorbent films.

Diglycidyl ethers are diepoxy polyols, commonly used as crosslinkers for the synthesis of sustainable materials (Shechter and Wynstra, 1956; Perez-Cantu et al., 2014; Abdulhameed et al., 2019; Cai et al., 2021; Culebras et al., 2021; LibreTexts, 2021). The specific polyol and chain length of polyol can be finely selected. Several DEs such as glycerol-DE (Issariyakul and Dalai, 2014), poly(ethylene glycol)-DE (Shelley, 2007), and poly(propylene glycol)-DE (Satapathy et al., 2018) can be synthesized from renewable materials, increasing their compatibility as crosslinkers for renewable polysaccharides.

Modified high viscosity carboxymethyl cellulose (CMC) is commonly used for the synthesis of superabsorbent materials (Kono, 2014; Capanema et al., 2018; Alam et al., 2019). CMC is a biodegradable (VanGinkel and Gayton, 1996), and water soluble (CPKelco, 2009) polyelectrolyte formed by reacting cellulose with monochloroacetate (MCA) (Joshi et al., 2015; Muhamad Parid et al., 2017; Fechter and Heinze, 2019). This reaction grafts carboxymethyl groups to the cellulose polymer, increasing its solubility in water to form a mucilaginous solution that can then be crosslinked (Gross et al., 1990). The resultant material properties are highly dependent on the grade of CMC used (Mw and DS), with high-viscosity CMC exhibiting the best gelling properties (CPKelco, 2009).

In this study, a series of superabsorbent films were synthesized by crosslinking CMC with four different DEs, including the novel poly(propylene glycol) diglycidyl ether. The structural and physiochemical properties of each polymer were compared in order to establish the effect of crosslinker type and chain length on material properties in the context of evaporative-dried, polysaccharide-based superabsorbent polymers.

## 2 Materials and methods

### 2.1 Materials

High-viscosity sodium carboxymethyl cellulose (DS = 0.9) with a molecular weight average of 700 kDa, four diglycidyl ethers (Table 1; Figure 1); glycerol diglycidyl ether (Mn 204 g/mol), poly(ethylene glycol) diglycidyl ether (Mn 500 g/mol), and poly(propylene glycol) diglycidyl ether (Mn 380 g/mol and Mn 640 g/mol), high purity D-(+)-cellobiose, and poly (ethylene glycol) Mn 400 g/mol) were purchased from Sigma-Aldrich. Sodium hydroxide (NaOH) pellets were purchased from Merck and made up into a 0.1 M solution to alkalize the CMC.

**TABLE 1:** Each diglycidyl ether investigated to crosslink carboxymethyl cellulose.

Crosslinker	ID	Number molecular weight average, Mn (g/mol)	Average chain length
Glycerol diglycidyl ether	GDE	204	1
Poly(propylene glycol) diglycidyl ether	PPG (380)DE	380	3
Poly(ethylene glycol) diglycidyl ether	PEGDE	500	5
Poly(propylene glycol) diglycidyl ether	PPG (640)DE	640	7.5

### 2.2 Polymer preparation

Polymers were separately prepared using one of the four diglycidyl ether crosslinkers in triplicate at five different stoichiometric concentrations (Table 2), including a pure CMC film as control, and 5 wt% poly(ethylene glycol) film as a plasticized but not crosslinked control. Each concentration was calculated per moles of anhydroglucose units (AGU) (Supplementary Information S1). The lowest concentration selected was the lowest at which the CMC would become insoluble after crosslinking. The synthesis method was derived from a method by Kundu and Banerjee (2019).

**TABLE 2:** Volumes of crosslinker added to 2.5 g of CMC in each replicate for the synthesis of the each CMC-DE film.

Crosslinker concentration	Volume of crosslinker (ml)			
(mol/mol AGU)	GDE	PPG (380)DE	PEGDE	PPG (640)DE
0.07	0.13	0.25	0.42	0.33
0.14	0.25	0.50	0.85	0.66
0.28	0.50	1.01	1.70	1.32
0.56	1.00	2.01	3.39	2.65
0.85	1.50	3.02	5.09	3.97

Deionized water (500 ml) was adjusted to pH nine by dropwise addition of 0.1 M NaOH solution. 2.5 g of sodium carboxymethyl cellulose (0.5 wt%) was then added and dissolved by rapid stirring overnight. Once fully dissolved, the solution was refrigerated at 4℃ for 24 h. This enabled the temperature sensitive diglycidyl ethers to be mixed into the solution before initiating the reaction.

Under rapid stirring, the determined volume of diglycidyl ether was pipetted into the CMC solution over 5 min. The mixture was stirred for a further 5 min before it was poured into beakers and placed in a water bath set to 70 ℃ for 2 h. Films were formed by pouring the crosslinked solutions into aluminium trays (152 mm × 181 mm) and oven dried at 35℃ .

### 2.3 Characterization

Fourier-transform infrared spectroscopy (FTIR) was performed on the 0.28 mol/mol films and CMC powder as a control. Spectra were recorded using a Cary 630 FTIR (Agilent Technologies) with a resolution of 4 $cm^{-1}$ and an average of 16 scans.

Evidence of crosslinking was tested for using ^13^C NMR with D-(+)-cellobiose as a model feedstock. Cellobiose, as the constitutive monomer of cellulose, is commonly used as a model compound for cellulose in reactions (Lu et al., 2017). Cellobiose was made into a 6% wt solution and alkalized to pH nine by addition of NaOH. PPG (640) DE was added to two samples at 0.07 mol/mol AGU and 0.85 mol/mol AGU concentrations, respectively. Which were then reacted in the same conditions and filtered using 0.2 μm syringe filters. NMR was performed using a Bruker AVIII 400 MHz spectrometer set to 1,000 scans and an acquisition time of 3 s. Unreacted PPG (640) DE and cellobiose were also analyzed as controls.

The pore volume distribution, bulk density, and porosity of CMC polymers were quantified using mercury intrusion porosimetry (MIP) with a Micromeritics Autopore IV 9500 V1.09 (Supplementary Information S2). Samples were washed with ethanol to remove residual polyol, before being cut into 1 cm × 1 cm squares and degassed at 50 ℃ for 24 h prior to analysis.

Scanning electron microscopy (SEM) images were acquired using a FEI Magellan FEG SEM microscope. Prior to imaging, samples were washed with absolute ethanol to remove residual polyol. Iridium (<1 nm) was applied to coat dried samples for preventing charging effects during imaging.

### 2.4 Free swelling capacity and water retention

The free swelling capacity and saline swelling capacity of each polymer were evaluated by immersion in deionized water and physiological saline (0.9% wt NaCl) respectively for 24 h and measuring the mass change. These solutions are common standards used for hydrogel swelling (Hossain et al., 2021; Karoyo and Wilson, 2021; Zainal et al., 2021). For swelling kinetics in deionized water, samples were weighed after 2, 10, 30, 180, 1,440, and 2,880 min. The wet mass was measured using a method by Zhang et al. (2020) with a slight modification; no vacuum was exerted on the materials due to the compressibility affecting water removal. Following this, samples were re-dried in an oven at 60℃ and the mass was measured. The free swelling capacity was calculated using Eq. 1:

$$\begin{array}{l} free\;swelling\;capacity,Q=\frac{m_{wet}-m_{dry}}{m_{dry}}, \end{array}$$ (1)

where $m_{wet}$ is the mass of wet sample and $m_{dry}$ is the final mass of the dry sample.

The water retention value (WRV) of the materials was determined following ISO 17190-6 with an adjustment by Cheng et al., (2010). Samples were immersed in deionized water for 48 h before them placing in polypropylene mesh bags suspended at the top of centrifuge tubes, and centrifuging at a relative centrifugal force (RCF) of 250 G for 3 min. The mass of the centrifuged sample was recorded, then the sample was dried and weighed to obtain the final dry mass. The WRV was calculated using Eq. 2:

$$\begin{array}{l} WRV=\frac{m_{c}-m_{dry}}{m_{dry}}, \end{array}$$ (2)

where $m_{c}$ is the mass of the centrifuged material.

Schott’s second-order kinetics model for semi-crystalline polymers was used to analyze swelling kinetics (Schott, 1992):

$$\begin{array}{l} \frac{dQ}{dt}=K_{is}{\left(\frac{Q_{\infty }-Q_{t}}{Q_{\infty }}\right)}^{2}, \end{array}$$ (3)

where $Q_{t}$ is the swelling rate at time, t, $K_{is}$ is the initial swelling rate constant, and $Q_{\infty }$ is the free swelling capacity at equilibrium. Correlations with this model indicate entropy-driven swelling (Schott, 1992). A linearized version of this model was applied to swelling kinetics data (Mohammad et al., 2017; Barajas-Ledesma et al., 2020):

$$\begin{array}{l} \frac{t}{Q_{t}}=\frac{1}{K_{is}}+\frac{1}{Q_{\infty }}t, \end{array}$$ (4)

### 2.5 Tensile testing

The crosslinked CMC films were cut into 140 mm × 15 mm rectangular strips. The samples equilibrated to conditions of 23℃ and 50% RH for 24 h. The minimum thickness of each sample was measured using a micrometer and used for tensile calculations. Tensile measurements were taken in 15 replicates for each sample using an Instron 5,965 universal Testing System following ASTM D638. The maximum tensile stress, strain at break, and Young’s modulus for each film were calculated by the system, with results of failed samples omitted.

Data was analyzed using Tukey’s one-way analysis of variance (ANOVA) with GraphPad Prism 9. Post hoc Duncan’s multiple range test (MRT) was performed using IBM IPSS Statistics 27 software to compare means for each crosslinker type.

## 3 Results

CMC was crosslinked with a series of DEs varying in concentration (0.07, 0.14, 0.28, 0.56, and 0.85 mol/mol AGU), resulting in four polymers: glycerol-CMC (G-CMC), poly (ethylene glycol)-CMC (PEG-CMC), 380 Mn poly (propylene glycol)-CMC (PPG (380)-CMC), and 640 Mn poly (propylene glycol-CMC (PPG (640)-CMC). These were characterized using FTIR, ^13^C NMR, SEM, and mercury intrusion porosimetry (MIP). Swelling and water retention properties were evaluated by immersion in deionized water and saline (0.9 wt% NaCl). Mechanical behavior was determined by tensile testing.

### 3.1 Chemical characterization

**FIGURE 2:**
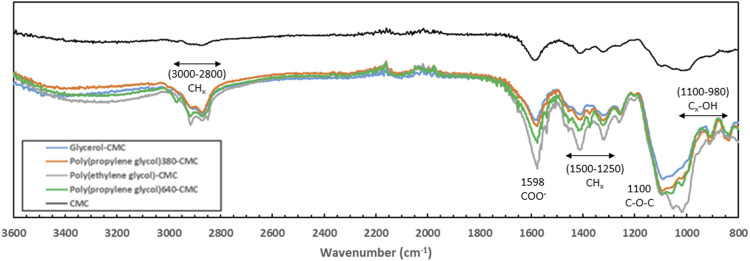
FTIR spectra for the family of diglycidyl ether crosslinked CMC films.

The FTIR spectra for CMC before and after crosslinking with the different diglycidyl ethers are shown in Figure 2. Due to the difference in sample form (powder or film) for the control and crosslinked polymers the peak intensities are not representative of concentration. Peaks between 980 cm^−1^ and 1,100 cm^−1^ are the C-OH bonds for different carbons in the 1–4, β -glucose monomers and the crosslinkers. The strong stretching vibration at 1,100 cm^−1^ for all crosslinked films is attributed to the ether linkages forming in the crosslinking reaction and present in the polyol by-product. The medium absorption bands between 1,250 cm^−1^ and 1,500 cm^−1^ result from the CH, CH_2_, and CH_3_ bending frequency vibrations. The additional peak at 1,390 $cm^{-1}$ for PPG (380)-CMC and PPG (640)-CMC spectra correlates with the presence of additional CH_3_ groups from the poly (propylene glycol) crosslinkers. The band at 1,598 cm^−1^ is the ionized carboxylate group present from the CMC (Capanema et al., 2018).

**FIGURE 3:**
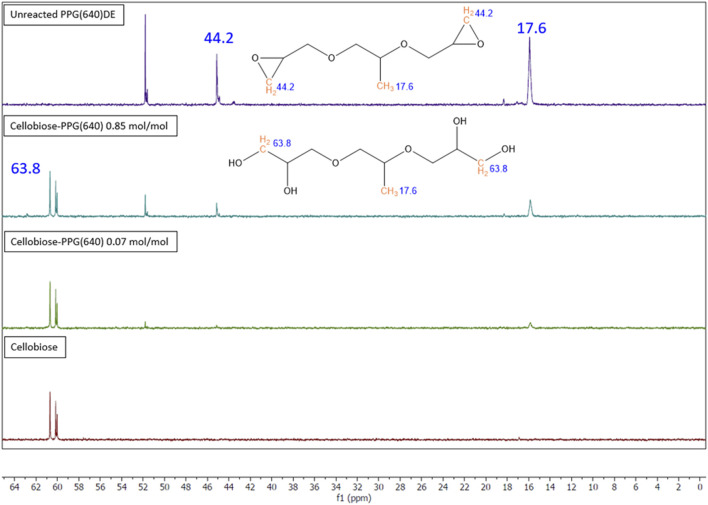
Solution 13C NMR spectra for crosslinked and control samples of cellobiose and PPG (640) DE with the unreacted and hydrolysed structures of PPGDE shown.

**FIGURE 4:**
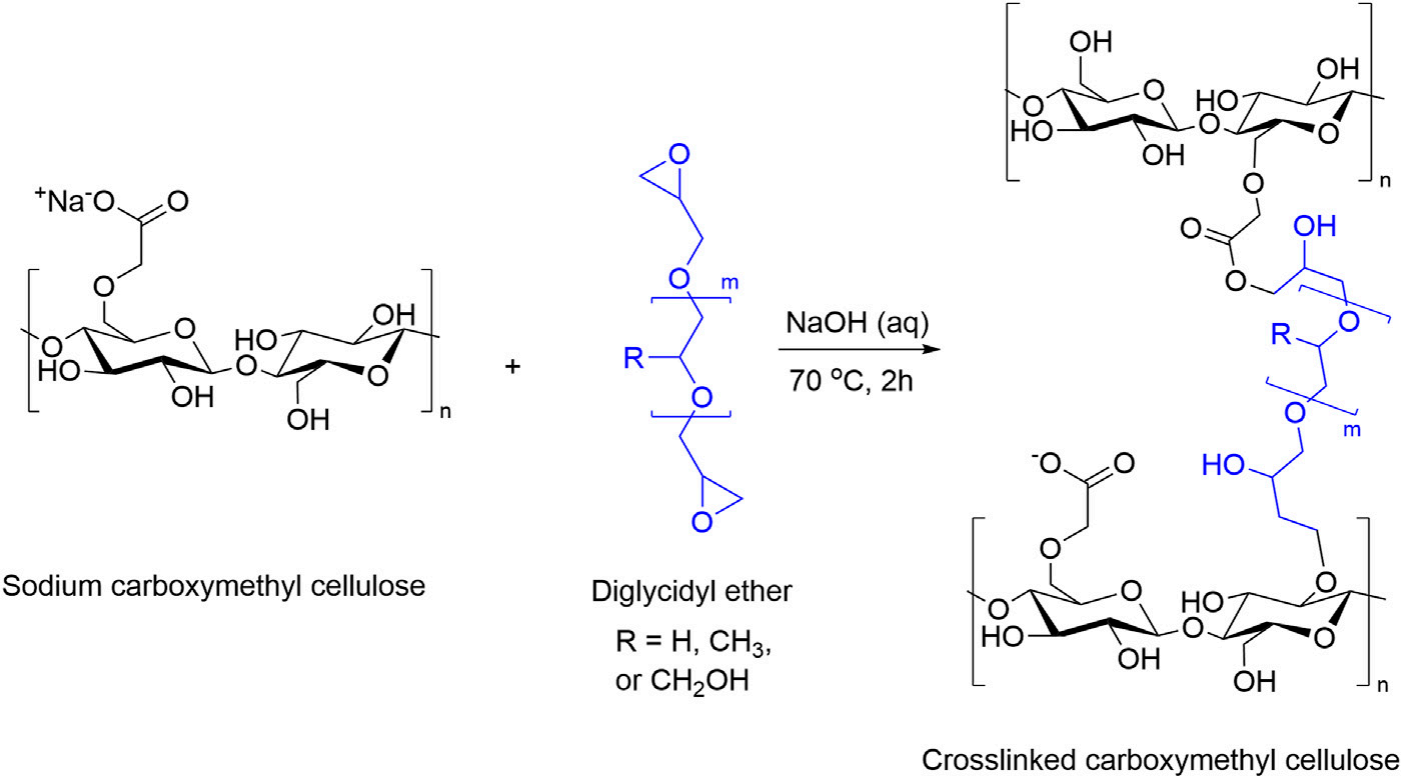
Proposed reaction scheme for the crosslinking of carboxymethyl cellulose with PEGDE (R = H), PPGDE (R = CH_3_), and GDE (R = CH_2_OH).

Solution 13C NMR spectra (Figure 3) show the crosslinking reaction between cellobiose and PPGDE. The peak at 17.6 ppm in the unreacted PPG (640)DE spectrum is the -CH_3_ carbon from PPG. The peak at 44.2 ppm is the CH_2_ carbon in the epoxy ring, and the peak at 63.8 ppm is the same carbon for hydrolyzed residual polyol. The ratio between these CH_3_ and CH_2_ peak integrals is evidence of unreacted/hydrolyzed epoxides in solution. This was calculated to be approximately 12% for each concentration, suggesting the remaining DEs are forming ether bonds under alkaline conditions. This gives a basis for the reaction scheme (Figure 4).

### 3.2 Structural morphology

Mercury intrusion porosimetry (MIP) was used to characterize the porosity, bulk density, and pore size distributions of each dry polymer. The bulk density varied between 850 kg/m^3^ and 1,050 kg/m^3^ and the porosity ranged between 33% and 42% (Supplementary Figure S1). The densities are reduced by the polyol additives and are lower than nanocellulose (CNF). The porosities are typical of low-temperature evaporation-dried CNF hydrogels (Barajas-Ledesma et al., 2020). The pore volume distributions show the presence of micro- and macro-pores for all materials, but only the G-CMC polymer exhibits the significant presence of nanopores.

**FIGURE 5:**
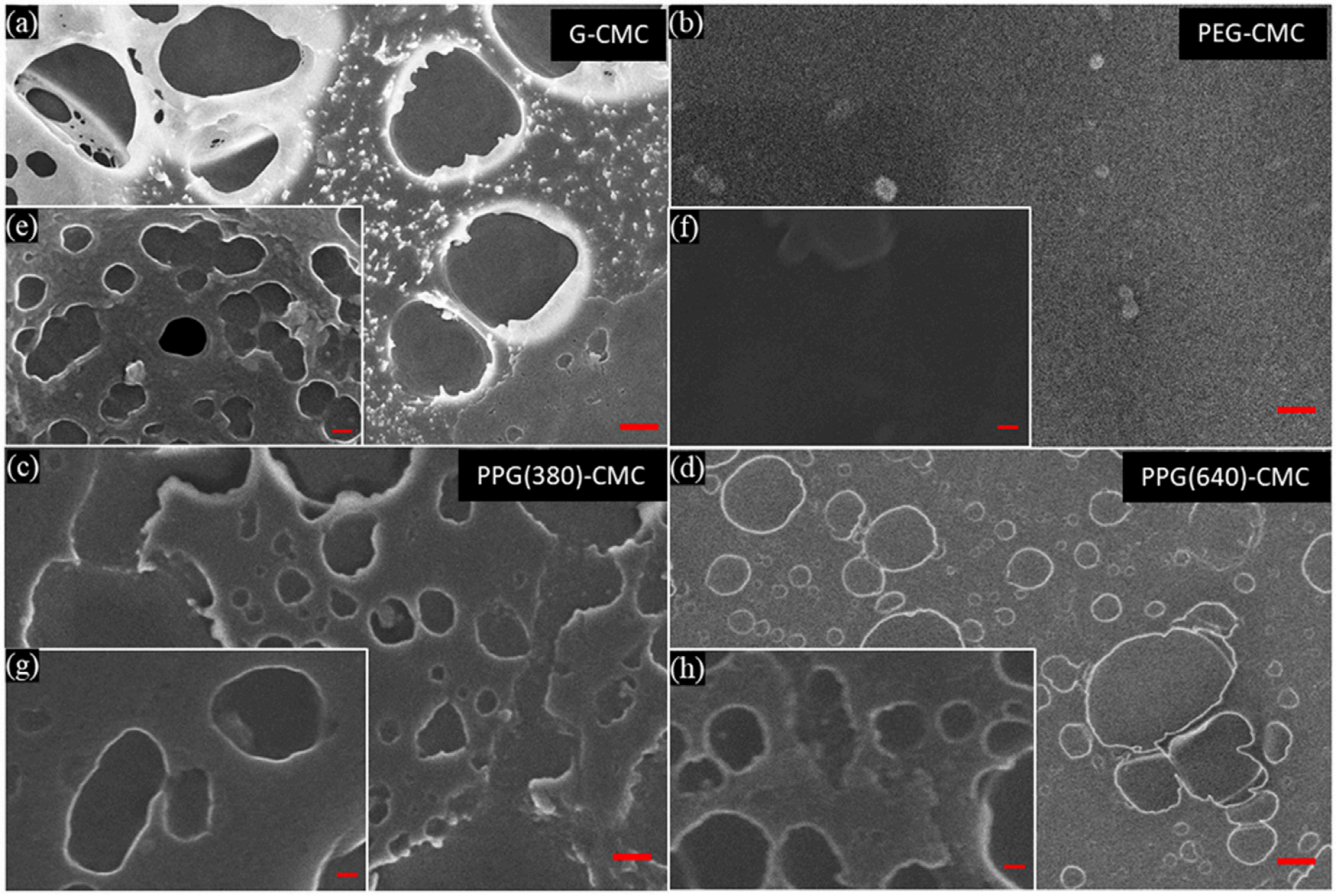
Scanning electron microscopy images of the oven dried, CMC-DE polymers, crosslinked at 70℃ for 2 h with a crosslinker concentration of 0.07 mol/mol. From **(A–D)** with 1 μm scale bars, G-CMC, PEG-CMC, PPG (380)-CMC, and PPG (640)-CMC respectively. **(E–H)** are corresponding magnified versions with 100 nm scale bars.

The morphology of the air-contacting surface of each 0.07 mol/mol polymer was imaged through SEM (Figure 5). Differing to MIP, the SEM images show the presence of both micropores and nanopores, the latter being overlooked in MIP analysis due to the higher proportion of micropores present in samples (Supplementary Figure S1). Images of the PEG-CMC polymer showed a flat surface with very few pores at all, indicating the residual hydrolyzed PEG formed a homogeneous layer on the surface of the material.

### 3.3 Swelling properties

**FIGURE 6:**
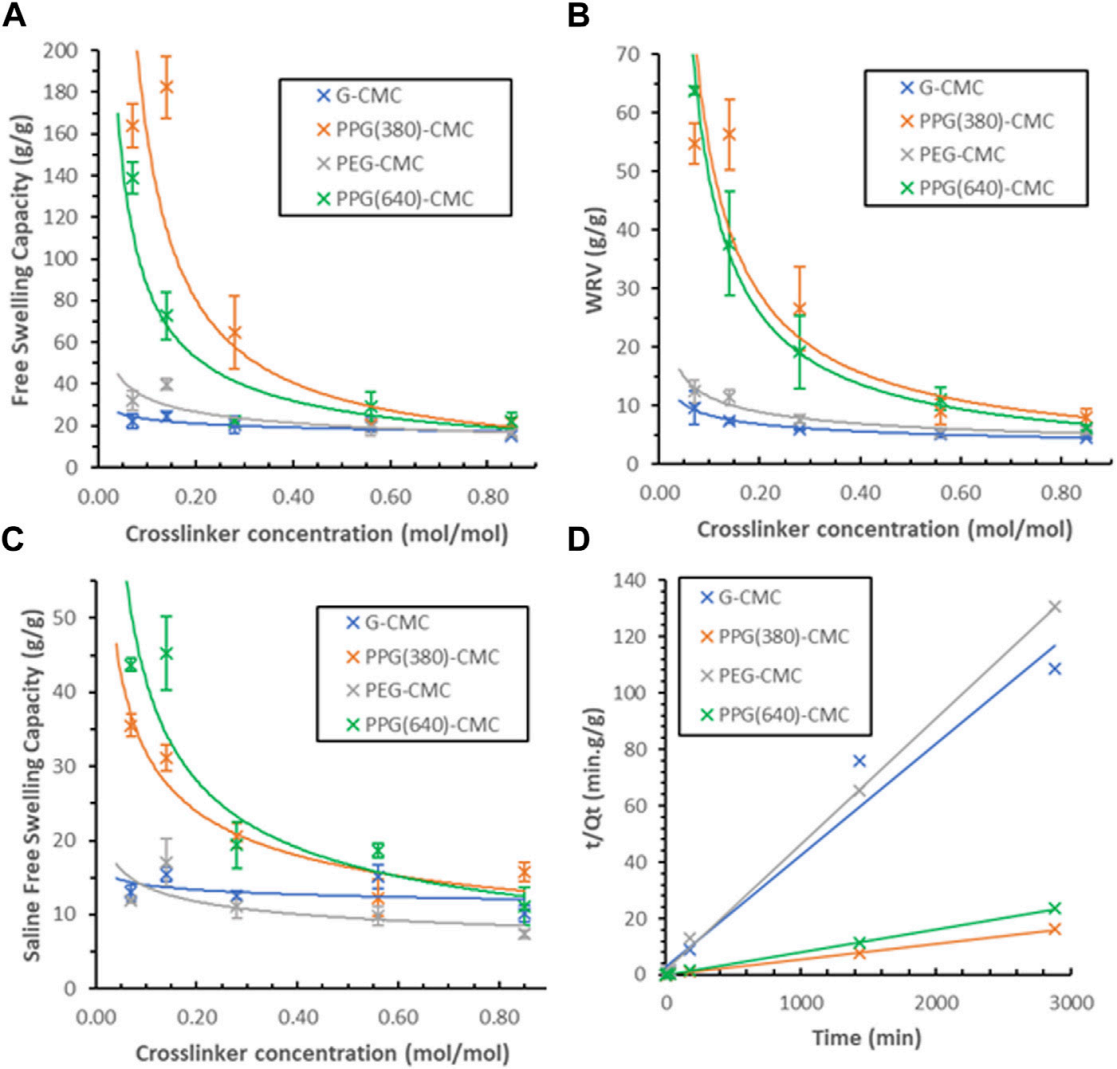
Swelling capacity of CMC-DE films; **(A)** Effect of the crosslinker concentration on the free swelling capacity of CMC materials in deionised water, **(B)** Effect of the crosslinker concentration on the water retention values for CMC hydrogels in deionised water, **(C)** Free swelling capacity of CMC materials in physiological saline (0.9%wt NaCl), **(D)** Schott’s model for inverse rate of swelling of each 0.07 mol/mol polymer.

The free swelling capacity of crosslinked films in deionized water (Figure 6A) revealed the 0.14 mol/mol PPG (380)-CMC polymer to have the highest swelling of 182±15 g/g. The 0.07 mol/mol PPG (640)-CMC polymer swelled to 139±8 g/g, and all G-CMC and PEG-CMC films showed poor swelling properties (lower than 40 g/g). The water retention value of crosslinked films (Figure 6B) correlates with their free swelling capacity, decreasing with increases in crosslinker concentration. The 0.07 mol/mol PPG (640)-CMC film had the highest WRV of 64±1 g/g. Swelling in 0.9 wt% NaCl physiological saline (Figure 6C) showed PPG-CMC has the highest degree of swelling at 36±2 g/g and 45±5 g/g for 380 Mn and 640 Mn, respectively. G-CMC and PEG-CMC films have lower swelling ratios of 15±2 g/g and 17±3 g/g each.

The swelling kinetics of 0.07 mol/mol polymers were measured in duplicate over 48 h (Figure 6D). A comparison between theoretical and actual swelling capacities at equilibrium (Q_theoretical_ vs. Q_actual_), as well as the initial swelling rate constant (K_iS_), swelling rate constant (K_S_), and the correlation coefficient (*R* ^2^) for each film is given in Supplementary Table S1. The greater slope observed with G-CMC and PEG-CMC materials indicate slower rates of swelling. The modelled theoretical swelling at equilibrium and the actual swelling at equilibrium are within 2 g/g for all samples $\left(R^{2}>0.96\right)$ .

**FIGURE 7:**
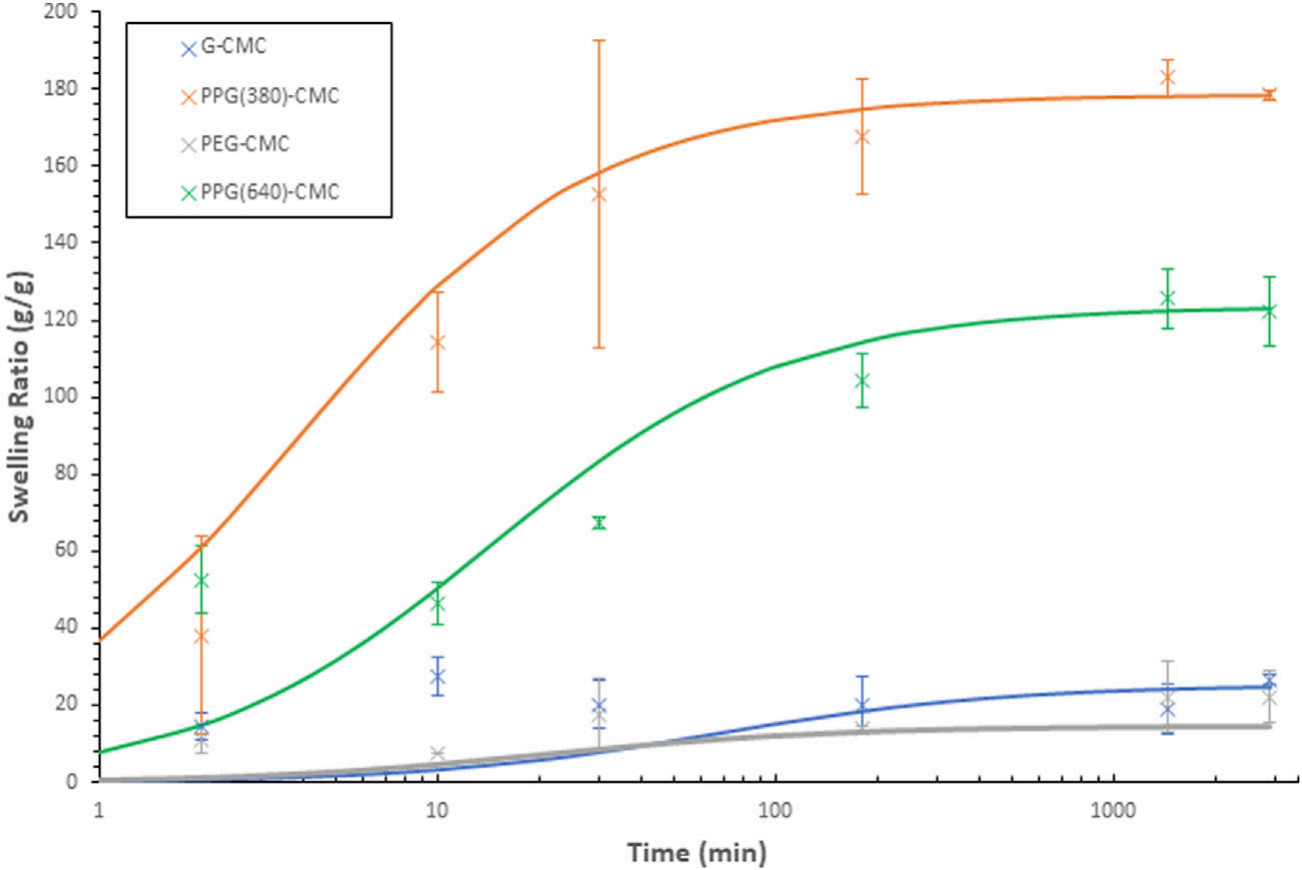
Swelling ratio of 0.07 mol/mol CMC-DE materials in deionised water over time.

The swelling curves are also plotted using Schott’s second-order equation Eq. 3 (Figure 7). The trend observed with the PPG-CMC films was a period of rapid swelling in the first 30 min which levelled off to a plateau over the 48 h. This is consistent with the swelling kinetics of cellulose-based materials (Mendoza et al., 2019a; Barajas-Ledesma et al., 2020; Hossain et al., 2021). The PEG-CMC and G-CMC films both reached their swelling equilibrium within 30 min, indicating water was only absorbed into the surface pores and swelling was inhibited by structural compaction (Schott, 1992).

### 3.4 Tensile testing

**FIGURE 8:**
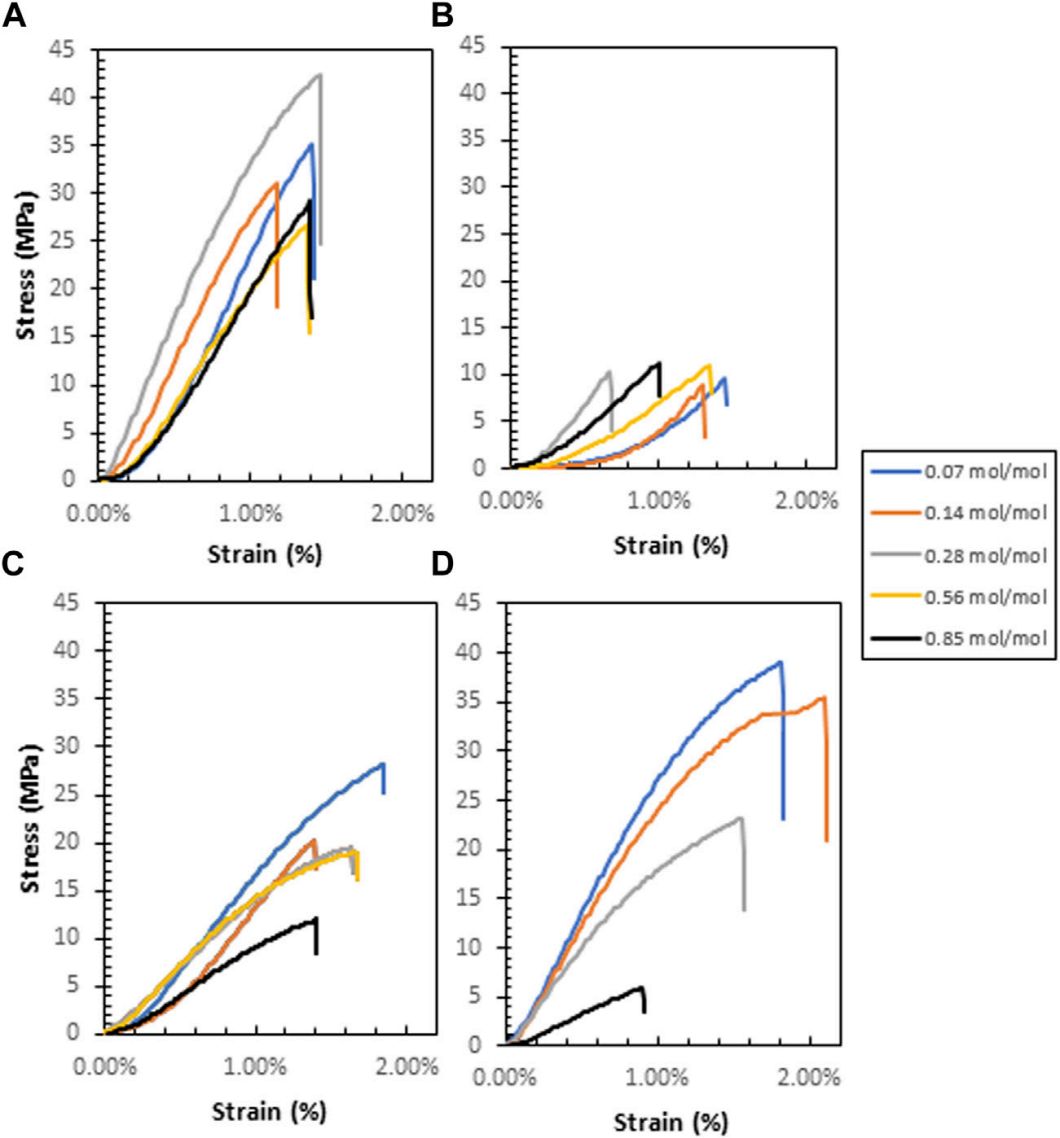
Nominal stress-strain curves for each polymer: **(A)**—G-CMC, **(B)**—PPG (380)-CMC, **(C)**—PEG-CMC, **(D)**—PPG (640)-CMC.

**FIGURE 9:**
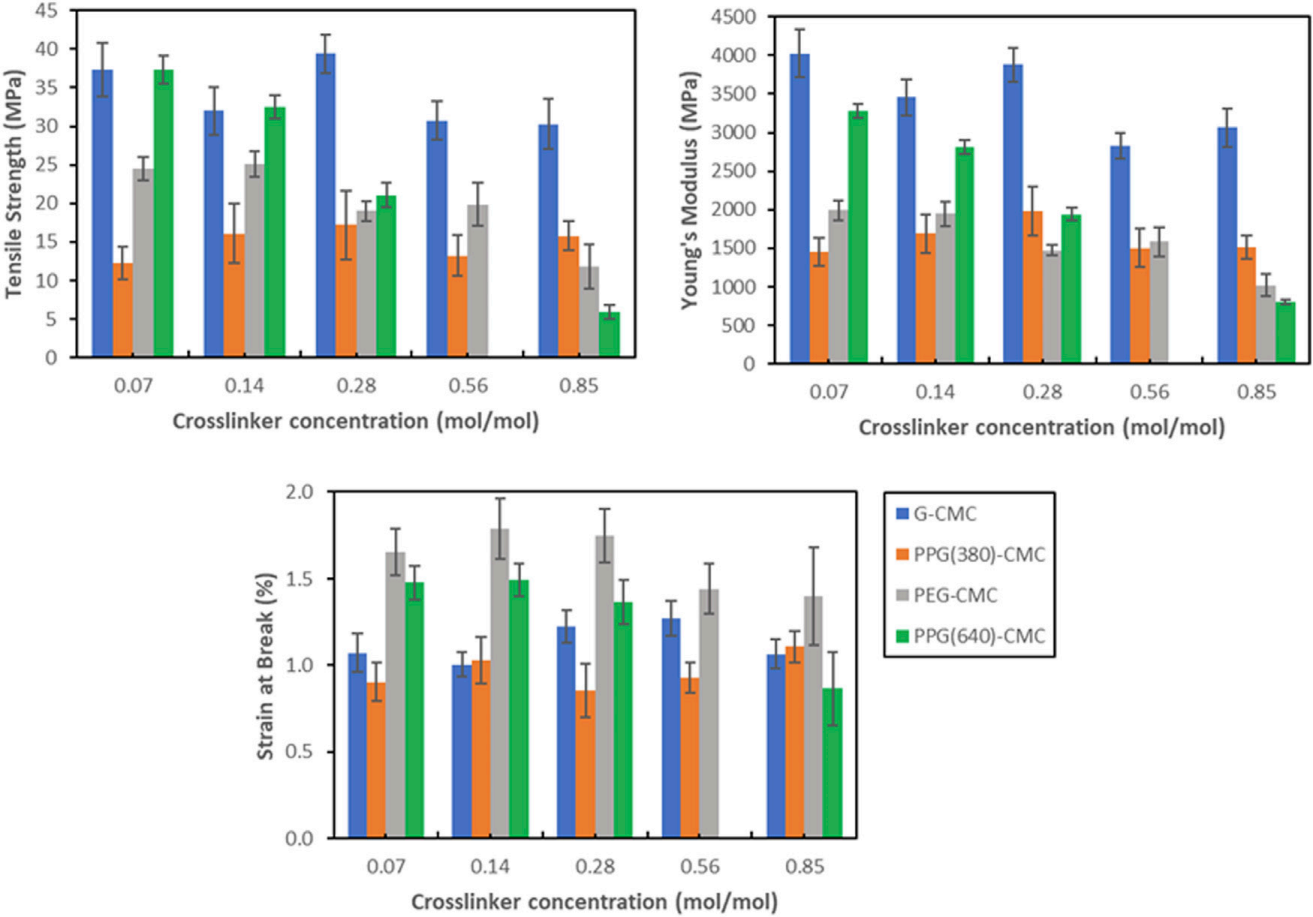
Tensile results for each CMC-DE polymer. Errors are given as standard error of mean.

Tensile measurements show the nominal stress-strain relationship for each polymer (Figure 8). G-CMC and PPG (640)-CMC are the strongest films, achieving maximum tensile strengths of 39±2 and 37±2 MPa, respectively (Figure 9). The pure CMC control film has a Young’s Modulus of 1400±200 MPa, a tensile strength of 18±2 MPa, and a strain at break of 1.6±0.3% . The plasticized 5% wt poly (ethylene glycol) film has a Young’s Modulus of 1,220 ± 60 MPa, a tensile strength of 12 ± 1 MPa, and a strain at break of 1.7 ± 0.2%. Only the PPG (640)-CMC films showed decreasing tensile strength with increasing crosslinker concentration. Increasing the crosslinker concentration also decreases the Young’s Modulus by plasticizing the materials.

## 4 Discussion

A series of diglycidyl ether-crosslinked carboxymethyl cellulose superabsorbent films (CMC-DE) were synthesized and characterized to establish the effect of crosslinker chain length and amphiphilicity on material properties.

### 4.1 Physicochemical characterization

NMR analysis showed a reduction in epoxy groups in solution, indicating ether bonds form during crosslinking. This is supported by literature that also demonstrated the formation of these bonds (Gross et al., 1990; Kono, 2014; Alam et al., 2019; Kundu and Banerjee, 2019). The apparent reduction in solubility observed between samples with increasing crosslinker concentration also supports this. NMR showed the presence of some unreacted diglycidyl ether which might react during drying, else indicating a higher pH or longer reaction time could be used to fully react all epoxides.

The pore size distributions for these materials (Supplementary Figure S1A) generally showed a constant distribution of pores between 1 μm and 100 μm , and the differences observed between different polymers were insignificant. The nanopores visible in SEM (Figure 5) are created as water permeates through the structure during drying (Pourjavadi et al., 2008), while the micropores visible in SEM and MIP are formed by the presence of residual polyol within the polymer structure; as water is evaporated, the residual polyol has a discontinuous phase transition with the crosslinked CMC and agglomerates in and on the surface of the polymer (Supplementary Figure S2). This also reduces the material bulk density (Barajas-Ledesma et al., 2020).

### 4.2 Swelling and water retention properties

The swelling performance of superabsorbent polymers is the primary factor affecting their use, and is the largest indicator of the materials response to the drying process. The highest free swelling capacity of 182 g/g was achieved by PPG-CMC polymer and is low compared to freeze-dried cellulose-based hydrogels (up to 725 g/g (Alam et al., 2019)). However, this is competitive with carboxylated evaporative-dried cellulose hydrogels (230 g/g) (Barajas-Ledesma et al., 2020).

#### 4.2.1 Factors affecting swelling

During drying, the material irreversibly transitions from a solution to an insoluble polymer film by the formation of interchain hydrogen bonds (Schott, 1992). Water can still permeate through the porous structure *via* capillary and osmotic forces (Eichler, Ramon, Cohen, Mizrahi) formed by hydrophilic interactions of water with polar functional groups, cleaving hydrogen bonds between adjacent CMC chains (Karoyo and Wilson, 2021). The maximum swelling ratio is restricted by the number of accessible hydrophilic groups and the equilibrium between forces governing mass transfer and hydrogen bonding. The crosslinked structure also retards swelling through the rigidity of covalent bonds preventing the structure to expand. Hence, there are three factors affecting the swelling of evaporative-dried polymers; hydrophilic group accessibility, structural rigidity formed by crosslinking, and chain agglomeration caused by hydrogen bonding.

Increasing the crosslinker concentration decreased swelling exponentially, irrespective of crosslinker type. Swelling is highest at the lowest crosslinker concentrations. This is attributed to the crosslinking density reducing the accessibility for hydrophilic groups and increasing the material rigidity. The crosslinking bonds between the DE and CMC reduce the number of accessible polar groups which facilitates swelling. Any increase in bond density also fixates the chains and suppresses expansion of the structure when swelling.

Initially it was believed that by increasing the hydrophilicity of the polymers, the hydrophilic group accessibility would increase and would maximize the swelling equilibrium. The relative hydrophobicity of each DE (C:O ratio) is 1.80, 2.25, 1.83, and 2.52 for GDE, PPG (380)DE, PEGDE, and PPG (640)DE, respectively. However, by increasing the hydrophilicity, the interchain hydrogen bond formation also increases, and this factor appears to dominate for evaporative-dried polymers. Evidence for this is shown by a decrease in swelling performance in the more hydrophilic polymers–G-CMC and PEG-CMC for the same crosslinker concentrations, even though the polymers have similar pore structures.

Therefore, contrary to freeze-dried superabsorbent polymers, increasing the hydrophobicity to an extent increases swelling performance. Hydrophobic groups present in the PPGDE crosslinkers reduce hydrogen bonding during drying, reducing the entropy required for water uptake, and possibly also generate more repulsion within the structure when swelling to increase the equilibrium (Guo et al., 2019; Kim et al., 2019). Another observation is that the additional hydrophobicity of PPG (640)-CMC polymers results in lower swelling than PPG (380)-CMC films. This suggests the chain length of crosslinker in combination with the degree of substitution (DS) of CMC can be adjusted to engineer the material properties, and that optimum properties exist at specific hydrophilic group (-COOH, -OH) to hydrophobic group (-CH_3_, -CH_2_) ratios within the structure. This will also be influenced by the CMC used; further optimization of the CMC chain length and DS is needed to maximize swelling.

This is further supported by the correlations with Schott’s swelling model. The good correlation observed for the PPG-CMC polymers (*R* ^2^ > 0.99) indicates that the reduction in interchain hydrogen bonding affects swelling to a greater extent than the decrease in hydrophilic groups, The PEG-CMC and G-CMC films reached swelling equilibrium within 30 min, indicating the dominating hydrogen bonding causes the materials to be wettable films rather than superabsorbent polymers.

Superabsorbent sensitivity to ionic solutions is critical for agricultural, personal care and biomedical applications. In these fields, the material is exposed to different concentrations of ions that restrict swelling (Pourjavadi et al., 2008). Normal physiological saline (0.9 wt% NaCl) (Tonog and AD, 2021) was thus selected for swelling experiments (Alam et al., 2019; Mendoza et al., 2019a). PPG (640)-CMC showed the highest rate of swelling in saline (45 g/g), while PPG (380)-CMC had a significantly lower swelling (36 g/g), even though it exhibited a higher free swelling capacity in deionized water. This indicates that the higher molecular weight crosslinker has a lower sensitivity to ionic solutions. These swelling values are slightly lower than cellulose-based hydrogels in physiological saline (50 g/g (Mendoza et al., 2019a) to 118 g/g (Alam et al., 2019). Swelling as determined in this study does not represent swelling performance in agricultural and biomedical applications as solutions in these systems consist of monovalent, divalent, and trivalent cations. Cations with increasing charge decrease swelling capacity due to an increase in salt sensitivity (Karoyo and Wilson, 2021).

The gravimetric retention of each material before and after a swelling cycle (Supplementary Figure S3; Supplementary Table S2) shows retentions ranging between 39% and 69%, dependent on the crosslinker concentration. Both residual polyol and soluble CMC removal contribute to these values.

### 4.3 Mechanical properties

Another effect of the crosslinkers on the evaporative-dried polymers is on mechanical properties. These control applications as alternatives to fossil fuel-derived films (Harunsyah and Fauzan, 2017). Diglycidyl ether crosslinking of CMC forms brittle films upon drying, with most polymers exhibiting improved properties compared to the control. This is because adding a crosslinker increases the degree of covalent bonding which increases rigidity, Young’s Modulus, and tensile strength of the material. However, this improvement in strength and rigidity behaves in tension with the competing addition of residual polyols that act as plasticizers, increasing the spacing between adjacent chains, thus reducing interchain secondary valence bond formation and increasing elasticity (Sinha-Ray et al., 2012). This is shown in the control film with 5% wt poly (ethylene glycol) plasticizer added, which had a decreased Young’s Modulus and tensile strength, in exchange for a slightly higher elongation. Therefore, the use of diglycidyl ether crosslinkers does not maximize tensile strength or elasticity due to the competing effects and the resultant materials have slightly higher yield strength and elasticity compared to the pure CMC film. Additionally, the residual polyols also have a phase separation effect with the CMC polymer, with agglomerations forming large pores within the structure of the material during evaporative drying that reduce the elongation at break and yield strength of the materials (Yang and Paulson, 2000), irrespective of the crosslinker concentration. This is because above a critical concentration, a decrease in compatibility between plasticizer and crosslinked polymer occurs (Vieira et al., 2011). This is most significant in the PPG (640)-CMC films where an increase in crosslinker concentration from 0.07 mol/mol to 0.85 mol/mol decreased tensile strength from 37 MPa to 6.0 MPa, Young’s modulus from 3.3 to 0.8 GPa, and strain at break from 1.5% to 0.9% (Figure 9; Supplementary Table S3).

Regardless of this inhibition of mechanical properties, the tensile performance of the films is comparable to PLA, a bioplastic used for biomedical and consumer applications (Farah et al., 2016; Castro-Aguirre et al., 2018). The glycerol-CMC films are the stiffest, exhibiting the highest tensile strength (39 MPa) and Young’s modulus (4.0 GPa). Washing the solution before drying can reduce the concentration of residual polyol in the films to further reduce the phase separation effect, increase tensile strength, and optimize mechanical properties.

## 5 Conclusion

A series of renewable polymers were prepared by crosslinking carboxymethyl cellulose (CMC) with four different diglycidyl ethers (DE) and evaporative-drying. The mechanical and swelling properties of these materials was measured to establish the effect of crosslinker chain length and amphiphilicity on resultant properties. We found no correlation of swelling performance with crosslinker chain length; instead, properties are dependent on the crosslinker hydrophobicity. More hydrophobic groups (CH_3_) in the novel PPG-CMC films decrease hydrogen bonding, reducing the entropy required for water uptake, and increasing electrostatic repulsions, thus achieving a maximum free swelling capacity of 182 g H_2_O/g. This indicates that the dominating factor affecting the swelling performance of evaporative-dried polysaccharide-based polymers is the amphiphilicity and not the hydrophilic group accessibility, as with freeze-dried polymers.

The plasticizing crosslinkers inhibited mechanical properties through a phase separation between residual hydrolyzed DE and crosslinked CMC. The G-CMC polymer exhibited the highest tensile strength of 39 MPa and a Young’s Modulus of 4.0 GPa, properties indicating potential applications in films. Modulating the crosslinking density and removing residual crosslinker can improve these materials for film applications.

Here, we established the mechanisms for swelling performance of evaporative-dried polymers and demonstrated the significance of the amphiphilicity of the material structure. Diglycidyl ethers play a dual-role as crosslinkers and plasticizers in the synthesis of evaporative-dried cellulose materials, reducing hornification to improve mechanical properties and swelling performance. This enables a new generation of materials for an array of value applications.

## Data Availability

The raw data supporting the conclusion of this article will be made available by the authors, without undue reservation.
