# Supplementary material for: Modulating superabsorbent polymer properties by adjusting the amphiphilicity
Source: Front Chem. 2022 Sep 13;10:1009616. doi: 10.3389/fchem.2022.1009616 (PMC9513321; doi:10.3389/fchem.2022.1009616)
Supplement: Supplementary file 1 [file DataSheet1.docx]

# Supplementary Information

## Calculations for volume of DE added to each film

Initially, the crosslinker was added in moles of crosslinker per moles of carboxylate, assuming the diglycidyl ethers only form ester linkages. However, further research proved this was not the case, so the crosslinker concentrations were converted to moles of crosslinker per moles of anhydroglucose (AGU).

The average molecular weight of carboxymethylated AGU was calculated using the degree of substitution of CMC as a ratio,

$${M_{w}}_{average}=\left( 1-DS \right)\times{M_{w}}_{anhydroglucose}+DS\times{M_{w}}_{CMC-monomer}$$

$$=\left( 1-0.9 \right)\times162+0.9\times242$$

$$=234\frac{g}{mol}$$

This was used to determine the moles of AGU in 2.5 g of CMC,

$$n_{anhydroglucose}=\frac{m}{{M_{w}}_{average}}$$

$$=\frac{2.5}{234}$$

$$=0.0107 mol$$

The volume of crosslinker added for the preparation of each film is given.

Using the crosslinkers densities of 1.229, 1.14, 1.14, and 1.14 g/mL for GDE, PPG(380)DE, PEGDE, and PPG(640)DE respectively, and the molecular weight of each crosslinker, these volumes are converted to moles. Dividing the moles of crosslinker by the moles of AGU calculated gives the concentration of crosslinker with respect to moles of AGU.

## Parameters for mercury intrusion porosimetry

- Measurements performed with Micromeritics Autopore IV 9500 V1.09.
- Samples were cut into small pieces (approximate 1 cm x 1 cm squares) to fit into the degassing holder and MIP sample holder.
- Samples degassed at 50°C for 24 hours before measurement.
- Analysis parameters: Contact angle 130°, Hg surface tension 485 dynes/cm, Hg density 13.5335 g/mL (i.e. the defaults in Micromeritics software)

## Schott’s Second-Order Kinetics

**Table SI:** Coefficients for Schott's 2^nd^ order model of swelling kinetics for each film.

|  | **Q_actual_** | **Q_theoretical_** | **K_is_** | **K_S_** | **R^2^** |
| --- | --- | --- | --- | --- | --- |
|  | **(g/g**$\mathbf{)}$ | **(g/g)** | **(g/g.min)** | **(g/g.min)** |  |
| **G-CMC** | 26.5 | 25.3 | 0.38 | 0.00059 | 0.968 |
| **PPG(380)-CMC** | 178.4 | 178.6 | 46.30 | 0.00145 | 1.00 |
| **PEG-CMC** | 13.8 | 14.6 | 0.71 | 0.00333 | 0.966 |
| **PPG(640)-CMC** | 122.2 | 123.5 | 8.53 | 0.00056 | 1.00 |

## Pore size distribution, bulk density, and porosity


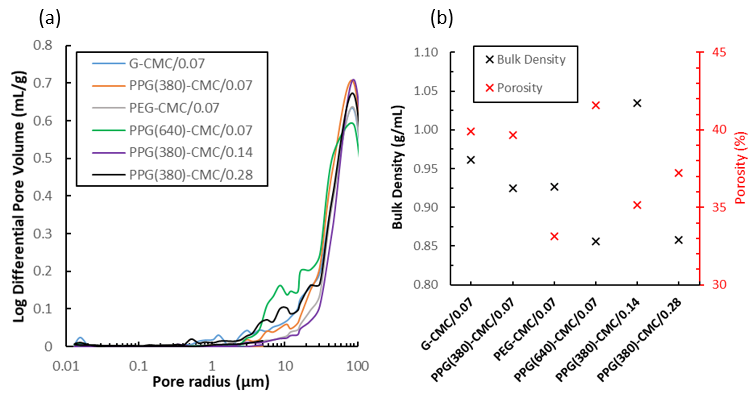


**Figure S1:** Mercury intrusion porosimetry for all 0.07 mol/mol materials and 0.14 mol/mol and 0.28 mol/mol PPG(380)-CMC; (a) Pore size distribution for different crosslinked polymers, (b) Bulk Density and Porosity of different crosslinked CMC-DE polymers.

## Low magnification SEM image


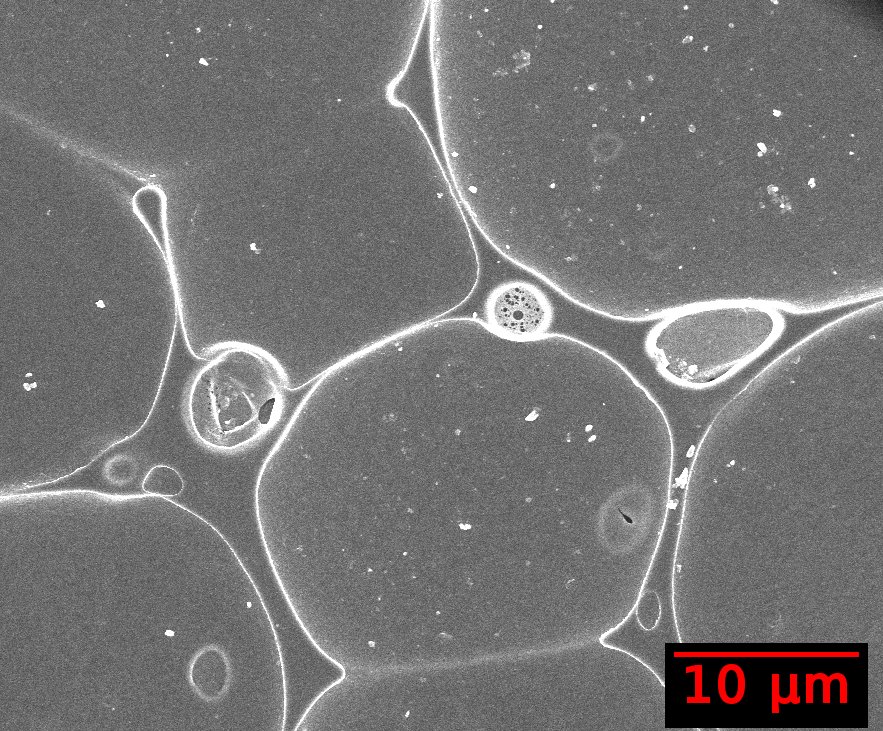


**Figure S2:** SEM image showing larger pores in PPG(380)-CMC 0.85 mol/mol polymer

## Mass retention after swelling and re-drying

The mass retention for each material after one swelling cycle is shown in **Figure S3**. These values are heavily influenced by the removal of residual polyol present in the polymers. Based on the mass of crosslinkers added, **Table SIII**, both CMC redissolving and polyol leaching contribute to these values. In polymers with higher percentages of crosslinker, the polyol leaching is the primary contribution, whereas in polymers with lower percentages of crosslinker, CMC redissolving is the primary contribution.

**Figure S3:** Percentage mass of each film retained after immersion in deionised water for 24 hours and re-drying

**Table SII:** Mass of crosslinker added to each polymer as a percentage of total sample weight

|  | **0.07** | **0.14** | **0.28** | **0.56** | **0.85** |
| --- | --- | --- | --- | --- | --- |
| **G-CMC** | 6.01% | 10.94% | 19.73% | 32.96% | 42.44% |
| **PPG(380)-CMC** | 10.23% | 18.57% | 31.53% | 47.82% | 57.93% |
| **PEG-CMC** | 13.08% | 23.13% | 37.57% | 54.72% | 64.42% |
| **PPG(640)-CMC** | 16.07% | 27.93% | 43.67% | 60.72% | 69.89% |

## Mechanical properties

**Table SIII:** Results of mechanical testing analysed using Tukey’s one-way ANOVA and Duncan’s post-hoc MRT. N.b. Data comparisons are for within each crosslinker type only.

| **Polymer** | **Crosslinker Concentration (mol/mol)** | **Young’s Modulus**  **(MPa)** | **Tensile Strength**  **(MPa)** | **Strain at Break (%)** |
| --- | --- | --- | --- | --- |
| **G-CMC** | 0.07 | 4000 ± 300^ab^ | 37 ± 3^a^ | 1.1 ± 0.1^a^ |
|  | 0.14 | 3500 ± 200^a^ | 32 ± 3^a^ | 1.00 ± 0.07^a^ |
|  | 0.28 | 3900 ± 200^a^ | 39 ± 2^a^ | 1.22 ± 0.09^a^ |
|  | 0.56 | 2800 ± 200^b^ | 31 ± 3^a^ | 1.3 ± 0.1^a^ |
|  | 0.85 | 3100 ± 300^b^ | 31 ± 3^a^ | 1.07 ± 0.08^a^ |
| **PPG(380)-CMC** | 0.07 | 1400 ± 200^a^ | 12 ± 2^a^ | 0.9 ± 0.1^a^ |
|  | 0.14 | 1700 ± 200^a^ | 16 ± 4^a^ | 1.0 ± 0.1^a^ |
|  | 0.28 | 2000 ± 300^a^ | 17 ± 4^a^ | 0.9 ± 0.2^a^ |
|  | 0.56 | 1500 ± 200^a^ | 13 ± 3^a^ | 0.90 ± 0.09^a^ |
|  | 0.85 | 1500 ± 100^a^ | 16 ± 2^a^ | 1.11 ± 0.09^a^ |
| **PEG-CMC** | 0.07 | 2000 ± 100^a^ | 25 ± 2^a^ | 1.7 ± 0.1^a^ |
|  | 0.14 | 1900 ± 200^a^ | 25 ± 2^a^ | 1.8 ± 0.2^a^ |
|  | 0.28 | 1470 ± 70^b^ | 19 ± 1^a^ | 1.7 ± 0.2^a^ |
|  | 0.56 | 1600 ± 200^ab^ | 20 ± 3^a^ | 1.4 ± 0.1^a^ |
|  | 0.85 | 1000 ± 100^c^ | 12 ± 3^b^ | 1.4 ± 0.3^a^ |
| **PPG(640)-CMC** | 0.07 | 3280 ± 90^a^ | 37 ± 2^a^ | 1.48 ± 0.09^a^ |
|  | 0.14 | 2810 ± 90^b^ | 32 ± 1^a^ | 1.49 ± 0.09^a^ |
|  | 0.28 | 1960 ± 80^c^ | 21 ± 2^b^ | 1.4 ± 0.1^a^ |
|  | 0.56 | - | - | - |
|  | 0.85 | 800 ± 30^d^ | 5.9 ± 0.9^c^ | - 1. 0.21^b^ |

## SEM image of stretched sample

An SEM image of a PEG-CMC film used for tensile testing was taken (**Figure S4**). This shows how the material behaves under stress. Material stretching is visible, probably resulting from tensile forces experienced during mechanical testing.


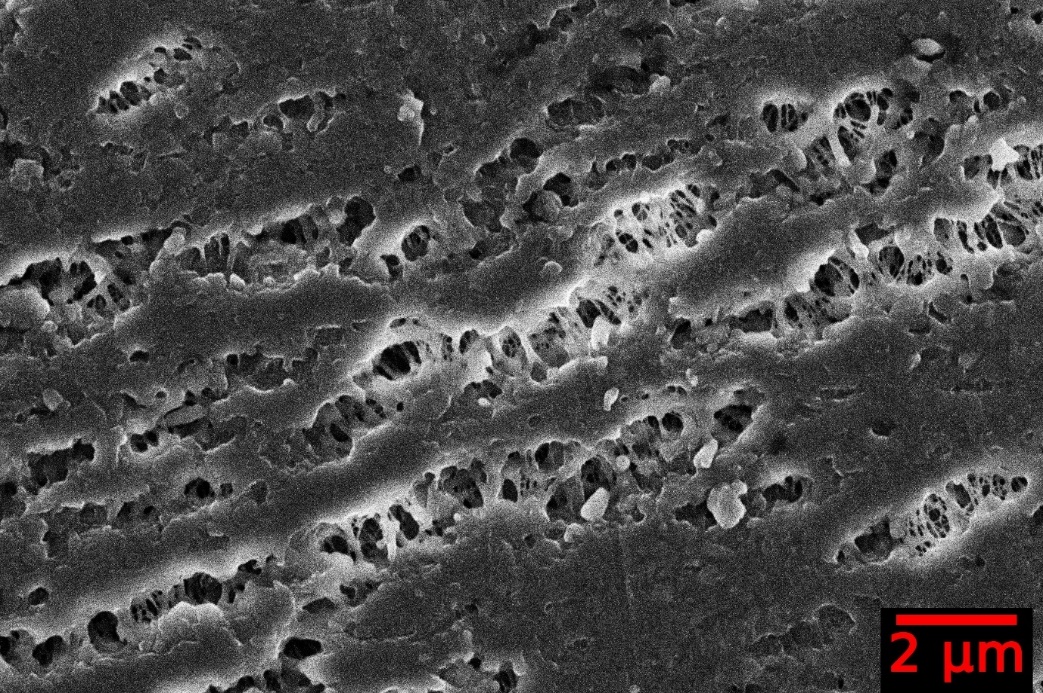


**Figure S4:** SEM image showing stretching in PEG-CMC 0.85 mol/mol polymer post-mechanical testing

## Solid-state NMR spectra

The crosslinking density of CMC-DE films synthesised using similar methodology has been quantified previously using solid state C13 NMR (SS NMR)20. To test this method for oven-dried samples, two samples of PPG(640)-CMC crosslinked with 0.07 and 0.28 mol/mol, respectively, were analysed using a Bruker AV 300 MHz solid-state spectrophotometer (SS 300) set to a spinning rate of 5 kHz.

The SS 13C NMR spectra for the 0.07 mol/mol and 0.28 mol/mol PPG(640)-CMC samples (**Figure S5**) both show peaks at 77 ppm and 119 ppm corresponding to the C6 and C1 carbons in cellulose. A broad peak observed for both spectra at 90 ppm includes peaks for the C2, C3, C4, C5, and C6 methyl carbons in CMC. The peaks at 193 ppm for both spectra is consistent for the carboxylate groups. However, the two spectra did not show the presence of the crosslinker. This is likely due to the low relative concentrations of DE and a crossover between the DE and CMC peaks causing the DE peaks to be hidden.


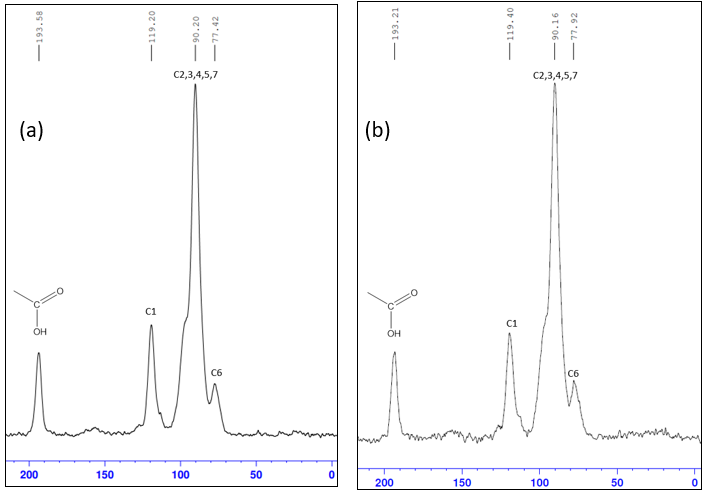


**Figure S5**: Solid state NMR spectra for (a) - PPG(640)-CMC/0.07 mol/mol and (b) - PPG(640)-CMC/0.28 mol/mol
